# Nonadherence to Cardiovascular Drugs Predicts Risk for Non-Arthritic Anterior Ischemic Optic Neuropathy: A Large-Scale National Study

**DOI:** 10.3390/jcm13164670

**Published:** 2024-08-09

**Authors:** Anan Hammud, Yosef. S. Haviv, Eyal Walter, Nir Amitai, Tomer Kerman, Samuel Leeman, Erez Tsumi

**Affiliations:** 1 Department of Ophthalmology, Soroka Medical Center, Ben-Gurion University of the Negev, Beer-Sheva 84101, Israel; ananhammoud@gmail.com (A.H.); eyal.walter@gmail.com (E.W.); samleeman95@gmail.com (S.L.); 2 Department of Nephrology, Soroka Medical Center, Ben-Gurion University of the Negev, Beer-Sheva 84101, Israel; havivy@bgu.ac.il; 3 Joyce & Irving Goldman Medical School, Ben-Gurion University of the Negev, Beer-Sheva 84101, Israel; amitaini@post.bgu.ac.il (N.A.); kerman@post.bgu.ac.il (T.K.); 4 Clinical Research Center, Soroka Medical Center, Faculty of Health Sciences, Ben-Gurion University of the Negev, Beer-Sheva 84101, Israel; 5 Medical School for International Health, Ben-Gurion University of the Negev, Beer-Sheva 84101, Israel

**Keywords:** NAION, nonadherence to drugs, cardiovascular disease

## Abstract

**Purpose**: While patients with cardiovascular comorbidities are at a higher risk for the occurrence of non-arteritic anterior ischemic optic neuropathy (NAION), it is unclear whether adherence to medication results in risk reduction. The purpose of this study was to investigate whether nonadherence to medical therapy for cardiovascular morbidity correlates with a higher risk for NAION when compared to patients with strict adherence. **Methods**: A retrospective case-control study was conducted among members of Clalit Health Services in Israel from 2001 to 2022. For each of the 757 NAION cases, three controls (totaling 2271 patients) were matched based on birth year and sex, with a propensity score analysis employed to adjust for a range of comorbidities. A patient was deemed nonadherent with medical treatment if their purchased quantity of medication was less than 60% of the prescribed annual dosage. Mixed models were used to evaluate exposure differences, and conditional logistic regression was applied, incorporating adjustments for socioeconomic status and ethnicity, to examine the impact of medication nonadherence on NAION risk. **Results**: A total of 3028 patients were included in the study; 757 patients with the diagnosis of NAION and 2271 in the matched control group. The average age of NAION patients was 69 ± 9 years and 55% were male. After adjustments for socioeconomic status and ethnicity, nonadherence to calcium channel blockers (CCBs) (odds ratio [OR]: 1.33, 95% confidence interval [CI]: 1.03–1.71) and anti-arrhythmic (OR: 5.67, 95% CI: 1.89–21.2) medications emerged as significant risk factors. Similarly, nonadherence to cardioprotective medications (OR: 1.46, 95% CI: 1.23–1.74) was also identified as a significant risk factor. **Conclusions**: Nonadherence to treatments for cardiovascular disease, specifically to medications known to improve prognosis, is associated with a higher risk for NAION.

## 1. Introduction

Non-arteritic anterior ischemic optic neuropathy (NAION) is the most common acute optic neuropathy in individuals above the age of 50 years. It ranks as the second leading cause of permanent vision loss related to optic nerve damage in adults, second only to glaucoma [1]. NAION is known to be a race-related disease, disproportionately affecting Caucasians compared to African American or Hispanic populations [2].

Typically, NAION manifests with sudden and painless vision loss in one eye, accompanied by a variable visual field defect, relative afferent pupillary defect, swollen and hyperemic optic disc and flame-shaped peripapillary retinal hemorrhages [3].

NAION poses a significant risk of irreversible vision loss, sometimes in both eyes. Patients who experience NAION in one eye face a 15–19% chance of a similar occurrence in the fellow eye within 5 years. Therefore, it is crucial to identify modifiable risk factors for NAION, particularly those amenable to medical treatment [4].

Although the direct pathophysiology of NAION is poorly understood, it is believed to result from hypoperfusion and ischemia of the optic nerve head, which results in edema that further reduces perfusion and subsequently damages the optic nerve fibers [5]. Several cardiovascular risk factors are also well-established risk factors for NAION, including hypertension, diabetes, smoking, elevated cholesterol levels, ischemic heart disease, and obstructive sleep apnea [6,7].

Despite the intuitive connection between cardiovascular risks and NAION occurrence, the association between strict adherence to cardiovascular drug therapy and its effect on NAION risk has not been investigated, particularly in large-scale studies. This study investigated whether lower adherence to prescribed medical therapy for cardiovascular morbidity correlates with an increased risk for NAION.

## 2. Materials and Methods

### 2.1. Study Population

This retrospective case-control study utilized electronic medical records from Clalit Health Services (CHS) in Israel, examining data from 1 January 2001 to 31 December 2022. Inclusion criteria were limited to individuals who had been members of CHS for at least one year, ensuring a reliable and consistent study population. As the largest health maintenance organization in Israel, CHS insures and provides medical services to approximately 4.8 million people, representing 51% of the Israeli population [8].

The case group consisted of patients diagnosed with NAION, identified by the International Classification of Diseases (ICD-9) code 377.41. To ensure accuracy in case selection and to exclude conditions that could mimic NAION, participants with a record of giant cell arteritis (ICD-9 code 446.5) or optic neuritis (ICD-9 code 377.30) within 30 days before or after the NAION diagnosis were omitted from the case group.

For each individual diagnosed with NAION, 3 control individuals were selected, matched on birth year and sex. As the existence of cardiovascular comorbidities is a risk factor for the occurrence of NAION, the matching process was further refined using a propensity score analysis to account for specific comorbidities, including peripheral vascular disease, myocardial infarction (MI), congestive heart failure, diabetes with and without complications, renal disease, metastasis, hemiplegia or paraplegia, malignancy, chronic pulmonary disease, and cerebrovascular disease. These comorbidities were coded according to ICD-9, as detailed in Supplementary Table S1.

### 2.2. Medication Compliance Assessment

Medication adherence was evaluated by analyzing each patient’s drug purchase history in the year preceding the NAION event, utilizing methodologies acceptable in the literature [9]. Patients were classified as nonadherent if they purchased less than 60% of their prescribed annual dosage for a given medication, based on the 55.5% rate reported by Adams et al. [10]. This nonadherence measure was individually calculated for specific medications, including aspirin, statins, ACE inhibitors/angiotensin II receptor blockers (ACE-I/ARBs), beta-blockers (BB), calcium channel blockers (CCB), antiarrhythmics, nitrates, doxazosin, and furosemide, identified by their ATC-5 classification codes, as detailed in Supplementary Table S2. These drugs were categorized into groups based on their therapeutic effects—either for medications that improve cardiac prognosis (aspirin, statins, ACE-I/ARBs, BB, CCB, and anti-arrhythmics) [11,12,13,14,15,16,17] or symptomatic drugs (furosemide, doxazocin, nitrates). Patients were considered non-adherent to a drug class if they failed to meet the adherence criteria for any single medication they had purchased within that group. This dual-layered analysis enabled a comprehensive evaluation of treatment compliance, both at the level of individual drugs and within defined drug classes.

### 2.3. Statistical Analysis

Continuous variables were described using means, medians, standard deviations (SDs), and ranges, while categorical variables were presented as frequencies and percentages. Univariate analysis was initially conducted to explore the differences between the case and control groups. Given the study’s matched design, mixed models were applied to evaluate differences in exposures between these groups. Subsequent multivariate analyses aimed to identify independent associations between NAION and medication adherence while adjusting for the potential confounders identified in the univariate analysis. Conditional logistic regression was employed to estimate odds ratios (ORs) with 95% confidence intervals (CIs), an approach particularly suited to the matched case-control study design, accounting for the correlation between matched pairs. All statistical tests were two-tailed, with significance set at a *p*-value less than 0.05. The analyses were performed using R software, version 4.3.1.

## 3. Results

A total of 3028 patients were included in the study, which included 757 patients diagnosed with NAION and 2271 patients who served as the matched control group. After propensity score matching, the standardized mean differences (SMDs) for all variables were lower than 0.1 (Supplementary Table S3).

Table 1 displays the demographic and clinical characteristics of the study population. The average age of patients with NAION was 69 ± 9 years, and 55% were male. There was a higher proportion of patients of Arab ethnicity in the case group (20%) compared to the control group (14%, *p* < 0.001). Differences were observed in socioeconomic status, with a greater percentage of NAION patients categorized as having low (case 20%, control 15%, *p* < 0.001) or high (case 22%, control 18%, *p* < 0.001) socioeconomic status. Of note, there was no trend association between the socioeconomic status and occurrence of NAION.

Table 2 displays the nonadherence rates for individual medications as well as for drug classes among the study participants, detailing both the number of patients who were expected to take each medication and the instances of nonadherence. For individual medications, significantly higher nonadherence rates were observed for CCBs in the case group (43%) vs. 37% in the control group (*p* = 0.027) and anti-arrhythmic agents (case: 89%, control: 60%, *p* = 0.001). Of note, these rates were derived from a low number of patients (Table 2), thus caution should be applied to this observation. Additionally, the nonadherence rate for the case group was notably higher for cardioprotective medications (67%) vs. control (59%, *p* < 0.001). Even in the absence of CCBs and anti-arrhythmic agents, the combination of cardioprotective medication with or without aspirin, the case group had a significantly higher nonadherence rate.

Table 3 presents the ORs for medication nonadherence, both unadjusted (crude OR) and adjusted (aOR) for ethnicity and socioeconomic status. Notably, nonadherence to CCBs (aOR: 1.33, 95% confidence interval [CI]: 1.03–1.71) and anti-arrhythmic medications (aOR: 5.67, 95% CI: 1.89–21.2) consistently emerged as significant risk factors in the case group compared to the control group before and after controlling for potential confounders. Similarly, nonadherence to the class of cardioprotective medications (OR: 1.46, 95% CI: 1.23–1.74) was also identified as a significant risk factor for both the crude and the adjusted model, within the case group relative to the control group.

## 4. Discussion

This study investigated whether nonadherence to prescribed medications for the treatment of cardiovascular morbidity can predict the occurrence of NAION. The findings suggest that poor adherence to cardiovascular medications, particularly those designed to improve cardiovascular prognosis, is linked to a 1.5-fold increased risk of NAION. To the best of our knowledge, no previous study has explored the relationship between adherence to drug therapy and the occurrence of NAION.

NAION primarily affects elderly patients with risk factors for atherosclerosis. Due to the nature of NAION’s vascular pathophysiology, it is not obvious that tight adherence to treatment of macrovascular disease risk factors can predict NAION [18]. For example, the UKPDS trial targeting optimal glycemic and blood pressure balance found that microvascular and macrovascular end points differed in their response to therapeutic efforts [19].

While NAION and drug adherence have not been the focus of previous studies, the association between the adherence to drugs for cardiovascular disease and the outcomes is well established. Kettani et al. found that high adherence to antihypertensive medications decreased the risk of cerebrovascular disease by 22% compared to lower adherence [20]. Psaty et al. reported that patients who were non-adherent to beta-blocker therapy (≤80% of dose; stopped medication during the preceding month) experienced a 4.5-fold increased risk of coronary heart disease [21]. A recent study by Chen et al. concluded that poor adherence to cardiovascular medications was associated with a considerable proportion of all cardiovascular disease events and even cardiovascular mortality. Specifically, adherence to angiotensin-converting enzyme inhibitors (ACEI)/angiotensin II receptor blockers (ARB) was associated with a relative risk (RR) of 0.90 for mortality, while adherence to statins showed an RR of 0.90 for cardiovascular events and an RR of 0.85 for mortality [22]. Moreover, adherence to CCB and statin medications was associated with lower risk of primary cardiovascular events [23]. Additionally, Mekonnen et al. demonstrated an association between drug nonadherence and outcomes of chronic kidney disease, including blood pressure, disease progression, and mortality [24].

Morbidity and the pathophysiological processes of the brain are largely parallel to the processes of the retina and optic nerve. NAION is considered equivalent to a brain stroke as the retina and optic nerve are essentially an extension of the brain. Interestingly, a recent meta-analysis published by Mafruhah et al. concluded that nonadherence tended to result in stroke and/or associated death (all pooled RR ≥ 1 and 95% CI did not include one) [25]. Furthermore, another study by Liu et al. used a dose-response analysis to demonstrate that a 20% improvement in adherence to any cardiovascular medication was associated with a 16% lower risk of stroke (RR: 0.84 and 95% CI). Precisely, Liu et al. noted that a 20% increase in adherence to anti-hypertensive and lipid-lowering medications was, respectively, linked to reduced risks of stroke by 17% (RR: 0.83, 95% CI) and 13% (RR: 0.87, 95% CI) [26].

Our findings align with several of the mentioned studies; we found that poor adherence to cardiovascular prognosis-improving medications was linked to a 1.5-fold increased risk of NAION. Specifically, this association was notable with calcium channel blockers (OR: 5.67, 95% CI) and antiarrhythmic medications (OR: 1.33, 95% CI).

To further support our hypothesis that cardiovascular risk modifier drugs may be significantly associated with fewer episodes of NAION, even in the absence of CCB and antiarrhythmic drugs, we tested whether a combination of drugs would also possibly be additively protective (despite individually failing) when combined into one group (Table 2). Furthermore, a combination of ACEI/ARBs, BB, and statins, with or without aspirin, was still significantly associated with fewer cases of NAION. Taken together, the combination of cardiovascular protective drugs may confer protection even when nonadherence to single drugs failed to predict NAION.

This result is plausible and may have several explanations, including the following: cardiovascular medications not only target hypertension and lower blood lipids but also exhibit pleiotropic effects. These include antioxidant and anti-inflammatory actions, improvement in vascular structure and function, modulation of cerebral hemodynamics, antithrombotic properties, and neuroprotective effects. These diverse mechanisms contribute to their preventative effects against cardiovascular events and stroke and could potentially influence the risk of NAION [27,28,29,30,31].

Previously, it was shown that physicians often ignore patient nonadherence to medications, thereby jeopardizing patient outcome. We believe that patients with NAION may benefit from their ophthalmologist paying attention to their adherence to some of the medications used to treat cardiovascular diseases, especially medications known to affect cardiovascular complications [32].

## 5. Strengths and Limitations

This study had several strengths, including its national scope (N = 3028), multi-center accessibility, and meticulous matching of parameters between the case and control patients to mitigate bias. However, it also had limitations. Its retrospective nature allowed us to establish associations but not causation. To further establish this connection, prospective studies may tap into databases of clinical trials and study whether our findings can be validated and thus improve the outcome of both ophthalmic and cardiovascular patients. Additionally, although we matched controls to include background comorbidities, we could not control for all potential confounding factors, which may include physicians’ decision-making regarding which agents to prescribe, adherence to novel anti-diabetic medications, or other unmeasured comorbidities (including co-existing ocular co-morbidities). Information on patient lifestyles was not available, and characteristics like smoking, diet, physical activity level, or obesity can also increase the risk for NAION. Lastly, the analysis classified patients based on whether they purchased a minimum of 60% of their prescribed annual dosage, but we could not determine whether the patient truly took all the medications purchased from the pharmacy. Thus, the current study may be regarded as hypothesis-generating for further prospective research, focusing on controlling cardiovascular risk factors and their impact on the risk of NAION disease.

## 6. Conclusions

In conclusion, we found that the occurrence of NAION was associated with lower rates of compliance to prescribed medications for cardiovascular disease, particularly those known as prognosis-improving drugs. Our results support the value of investing more effort into improving patients’ medication adherence, especially those relevant in NAION prevention. This task is also important to mitigate the risk of recurrent events, particularly in the fellow eye.

When addressing polypharmacy in patients at risk for NAION, priority should be given to medications with a positive impact on cardiovascular disease. This approach underscores the importance of managing cardiovascular risk factors to reduce the risk of NAION and its associated complications. Additionally, it is beneficial for patients with NAION to be referred to a specialist in internal medicine or a cardiologist for a thorough evaluation of their cardiovascular risk factors.

## Figures and Tables

**Table 1:** Baseline Characteristics After 1:3 Propensity Score Matching.

Characteristic	Total N = 3028	Case N = 757	Control N = 2271	*p*-Value
Age				>0.9
Mean ± SD	69 ± 9	69 ± 9	69 ± 9	
Median (IQR)	70 (63, 76)	70 (63, 76)	70 (63, 76)	
Range	45–90	45–90	45–90	
Male sex, n (%)	1652 (55)	413 (55)	1239 (55)	>0.9
Ethnicity, n (%)				<0.001
Jewish	2480 (82)	585 (77)	1895 (83)	
Arab	459 (15)	151 (20)	308 (14)	
Other	89 (2.9)	21 (2.8)	68 (3.0)	
Socioeconomic score, n (%)				<0.001
Low	469 (16)	141 (20)	328 (15)	
Medium	1867 (65)	422 (59)	1445 (67)	
High	550 (19)	155 (22)	395 (18)	
Comorbidities, n (%)				
Peripheral vascular disease	349 (12)	256 (11)	93 (12)	0.5
Myocardial infarction	338 (11)	242 (11)	96 (13)	0.13
Congestive heart failure	327 (11)	253 (10)	92 (12)	0.2
Diabetes with complications	491 (16)	363 (16)	128 (17)	0.6
Diabetes without complications	1775 (59)	1335 (59)	440 (58)	0.7
Renal disease	518 (17)	382 (17)	136 (18)	0.5
Malignancy	361 (12)	263 (12)	98 (13)	0.3
Metastasis	19 (0.6)	16 (0.7)	3 (0.4)	0.3
Hemiplegia or paraplegia	80 (2.6)	60 (2.6)	20 (2.6)	>0.9
Chronic pulmonary disease	878 (29)	659 (29)	219 (29)	>0.9
Cerebrovascular disease	804 (27)	601 (26)	203 (27)	0.8

**Table 2:** Medication Nonadherence.

Drugs	Case, N = 757			Control, N = 2271			*p*-Value
	Treated Patients (n)	Non-Adherent Patients (n)	Nonadherence Rate (%)	Treated Patients (n)	Non-Adherent Patients (n)	Nonsdherence Rate (%)	
Specific Drugs							
Aspirin	603	187	31	1355	431	32	0.7
Statins	535	183	34	1573	516	33	0.6
ACE-I/ARBs	525	214	41	1401	514	37	0.1
BB	379	130	34	1025	324	32	0.3
CCBs	358	153	43	862	310	37	0.027
Anti-Arrhythmic	38	34	89	50	30	60	0.001
Nitrates	93	57	61	190	110	58	0.6
Doxazosin	84	27	32	193	70	36	0.5
Furosemide	168	78	46	338	151	45	0.7
Therapeutic Drug Groups							
Cardioprotective drugs	753	507	67	2242	1312	59	<0.001
Non-cardioprotective drugs	252	143	57	574	304	53	0.3
Aspirin, Statin, ACE-I/ARB and BB	746	457	61	2188	1214	55	0.006
Statin, ACE-I/ARB and BB	698	380	54	2085	1036	50	0.03

Abbreviations: ACE-I, angiotensin-converting-enzyme inhibitors; ARBs, angiotensin receptor blockers; BB, Beta blockers; CCBs, Calcium channel blockers. Notes: Cardioprotective drugs include aspirin, statins, ACE-I/ARBs, BB, CCB, and anti-arrhythmic drugs. Non-cardioprotective drugs include nitrates, doxazosin and furosemide.

**Table 3:** Odds Ratios for NAION in Non-Adherent Versus Adherent Patients, Crude and Adjusted rates for Socioeconomic Status and Ethnicity.

Drugs	Crude Model			Adjusted Model		
	OR	95% CI	*p*-Value	OR	95% CI	*p*-Value
Specific Drugs						
Aspirin	0.96	0.78, 1.18	0.7	0.93	0.75, 1.15	0.5
Anti-Arrhythmic	5.67	1.89, 21.2	0.004	9.69	2.57, 51.0	0.002
Statins	1.06	0.86, 1.31	0.6	1.08	0.87, 1.34	0.5
ACE-I/ARBs	1.19	0.97, 1.46	0.1	1.18	0.96, 1.46	0.12
BB	1.13	0.88, 1.45	0.3	1.1	0.85, 1.42	0.5
CCBs	1.33	1.03, 1.71	0.027	1.32	1.02, 1.72	0.034
Doxazosin	0.83	0.48, 1.42	0.5	0.73	0.41, 1.28	0.3
Furosemide	1.07	0.74, 1.56	0.7	1.10	0.75, 1.60	0.6
Nitrates	1.15	0.70, 1.92	0.6	1.13	0.67, 1.92	0.6
Therapeutic Drug Groups						
Cardioprotective Drugs	1.46	1.23, 1.74	<0.001	1.42	1.19, 1.71	<0.001
Non-Cardioprotective Drugs	1.17	0.87, 1.57	0.3	1.15	0.85, 1.57	0.4
Aspirin, Statin, ACE-I/ARB and BB	1.27	1.07, 1.50	0.006	1.24	1.04, 1.47	0.018
Statin, ACE-I/ARB and BB	1.21	1.02, 1.44	0.03	1.20	1.00, 1.43	0.044

Abbreviations: ACE-I, angiotensin-converting-enzyme inhibitors; ARBs, angiotensin receptor blockers; BB, beta blockers; CCBs, calcium channel blockers; Notes: Cardioprotective drugs include aspirin, statins, ACE-I/ARBs, BB, CCB, and anti-arrhythmic. Non-cardioprotective drugs include nitrates, doxazosin and furosemide.

## Data Availability

The data files used for the present study are not publicly available according to the privacy regulations of Clalit Health Services. Data were extracted via the CHS Data Sharing Platform, powered by MDClone (accessed on 14 March 2024). This platform uses advanced algorithms to deidentify electronic medical record data, ensuring both data integrity and patient privacy. The authors did not have access to information that could identify individual participants during or after data collection.

## Supplementary Materials

The following supporting information can be downloaded at: https://www.mdpi.com/article/10.3390/jcm13164670/s1. Table S1: ICD-9 Codes; Table S2: ATC5 codes; Table S3: Baseline Characteristics and Standardized Mean Differences of Study Population After 1:3 Propensity Score Matching.
