# Supplementary material for: Nonadherence to Cardiovascular Drugs Predicts Risk for Non-Arthritic Anterior Ischemic Optic Neuropathy: A Large-Scale National Study
Source: J Clin Med. 2024 Aug 9;13(16):4670. doi: 10.3390/jcm13164670 (PMC11355576; doi:10.3390/jcm13164670)
Supplement: Supplementary file 1 [file jcm-13-04670-s001.zip › jcm-3142434-supplementary.pdf]

Supplementary Material

SupplementaryTable S1. ICD-9 Codes

Diagnosis description	ICD-9 Codes
Peripheral vascular disease	437.3, 440.x, 441.x, 443.1–443.9, 447.1, 557.1, 557.9
Myocardial infarction	410.x, 412.x
Congestive heart failure	398.91, 402.01, 402.11, 402.91, 404.01, 404.03, 404.11, 404.13, 404.91, 404.93, 425.4–425.9, 428.x
Diabetes with complications	250.4–250.7
Diabetes without complications	250.0–250.3, 250.8, 250.9
Renal disease	403.01, 403.11, 403.91, 404.02, 404.03, 404.12, 404.13, 404.92, 404.93, 582.x, 583.0–583.7, 585.x, 586.x, 588.0
Malignancy	140.x-172.x, 174.x-195.8, 200.x-208.x, 238.6
Metastasis	196.x-199.x
Hemiplegia or paraplegia	334.1, 342.x, 343.x, 344.0–344.6, 344.9
Chronic pulmonary disease	416.8, 416.9, 490.x-505.x, 506.4, 508.1, 508.8
Cerebrovascular disease	362.34, 430.x-438.x
Peptic ulcer disease	531.x-534.x
Moderate/severe liver disease	456.0–456.2, 572.2–572.8
Mild liver disease	570.x, 571.x, 573.3, 573.4, 573.8, 573.9
Dementia	290.x, 294.1, 331.2

Supplementary Table S2. ATC5 codes

Group	ATC-5 Codes
Aspirin	N02BA01
	A01AD05
	B01AC06
Nitrates	C01DA08
	C01DA14
	C01DA02
	C05AE01
Statins	C10AA01
	C10AA03
	C10AA05
ACE-I/ARBs	C09AA01
	C09AA02
	C09AA05
	C09AA08
	C09CA01
	C09CA03
Beta blockers	C07AB02
	C07AB03
	C07AB07
	C07AG02
Calcium channel blockers	C08CA01
	C08CA02
	C08CA05
	C08CA13
	C08DA01
Doxazosin	C02CA04
Furosemide	C03CA01
Anti-arrhythmic	C01BD01
	C01BC03
	C01BC04

ACE-I, angiotensin-converting-enzyme inhibitors; ARBs, angiotensin receptor blockers

Supplementary Table S3. Baseline Characteristics and Standardized Mean Differences of Study Population After 1:3 Propensity Score Matching

**Characteristic**	**Overall**<br>**N = 3028**	**Case**<br>**N = 757**	**Control**<br>**N = 2271**	**Standardized**<br>**Mean Difference**
Age				0
Mean ± SD	69 ± 9	69 ± 9	69 ± 9	
Median (IQR)	70 (63, 76)	70 (63, 76)	70 (63, 76)	
Range	45–90	45–90	45–90	
Male sex, n (%)	1652 (55)	413 (55)	1239 (55)	0
Comorbidities, n (%)				
Peripheral vascular disease	349 (12)	256 (11)	93 (12)	0.5
Myocardial infarction	338 (11)	242 (11)	96 (13)	0.13
Congestive heart failure	327 (11)	253 (10)	92 (12)	0.2
Diabetes with complications	491 (16)	363 (16)	128 (17)	0.6
Diabetes without complications	1775 (59)	1335 (59)	440 (58)	0.7
Renal disease	518 (17)	382 (17)	136 (18)	0.5
Malignancy	361 (12)	263 (12)	98 (13)	0.3
Metastasis	19 (0.6)	16 (0.7)	3 (0.4)	0.3
Hemiplegia or paraplegia	80 (2.6)	60 (2.6)	20 (2.6)	>0.9
Chronic pulmonary disease	878 (29)	659 (29)	219 (29)	>0.9
Cerebrovascular disease	804 (27)	601 (26)	203 (27)	0.8
